# Supplementary material for: MetaRibo-Seq measures translation in microbiomes
Source: Nat Commun. 2020 Jun 29;11:3268. doi: 10.1038/s41467-020-17081-z (PMC7324362; doi:10.1038/s41467-020-17081-z)
Supplement: Supplementary file 10 — Supplementary Data 7 [file 41467_2020_17081_MOESM10_ESM.zip › File2/Confidence_High_Taxonomy/203832_out.krona.html]

Javascript must be enabled to view this page.

members
magnitude
magnitudeUnassigned
count
unassigned
taxon
rank

203832\_out

3

2
3
superkingdom

201174
3
phylum

3
1760
class

3
2037
order

family
3
2049

3
1654
genus

272548
1
species

SRS149237\_contig\_number\_contig-100\_23113.23114


SRS015692\_contig\_number\_4225SRS016331\_contig\_number\_contig-100\_4462.65890
species
2
461393
